# Chemistry

**Published:** 1846-12

**Authors:** 


*Chemistry.*—The equivalents of silver, potassium, and chlorine have been hitherto calculated from the results of the analysis of the chlorate of potass. M. Maumené employs a new method, based upon the decomposition of the oxalate and acetate of silver, and finds the following equivalents, which differ only in a slight degree from those heretofore adopted:—Silver, 1390·42; chlorine, 442·04; and potassium, 487·78.—*Ibid.*

